# Incidence of Nausea and Vomiting After Fast-Track Anaesthesia for Heart Surgery

**DOI:** 10.21470/1678-9741-2018-0040

**Published:** 2018

**Authors:** Emad Mohamed Hijazi, Hayel Edwan, Nabil Al-Zoubi, Hadi Radaideh

**Affiliations:** 1 Faculty of Medicine, Jordan University of Science and Technology, Irbid, Jordan.; 2 Queen Alia Heart Institute, Amman, Jordan.; 3 General Surgery-Vascular, King Abdullah University Hospital and Jordan University of Science and Technology, Irbid, Jordan.; 4 General Surgery, King Abdullah University Hospital and Jordan University of Science and Technology, Irbid, Jordan.

**Keywords:** Anesthesia, Cardiac Procedures, Vomiting, Nausea, Postoperative Nausea and Vomiting

## Abstract

**Objective:**

The aim of this study was to evaluate the incidence of postoperative nausea and vomiting (PONV) after fast-track cardiac anesthesia (FTCA) in the first 24-48 hours in the cardiac intensive care unit (CICU) after open-heart surgery, risk factors for PONV and its influence on CICU length of stay.

**Methods:**

A prospective observational study from January 1, 2013 to the end of December 2015 was performed in the CICU of a university hospital in the north of Jordan and Queen Alia Heart Institute, Amman, Jordan. Three hundred consecutive patients undergoing fast-track cardiac anesthesia in elective cardiac surgery were enrolled in the study. Nausea and vomiting were assessed after tracheal extubation, which was performed within 6-10 hours after surgery and during the first 24-48 hours in the CICU. Metoclopramide 10 mg intravenously was used as the initial antiemetic drug, but ondansetron 4 mg intravenously was also used as second line of management.

**Results:**

Nausea was reported in 46 (15.3%) patients, and vomiting in 31 (10.3%). Among females, 38 (33.9%) patients developed nausea and 20 (17.9%) developed vomiting. Among males, 8 (4.3%) patients developed nausea and 11 (5.9%) developed vomiting.

**Conclusion:**

PONV are relatively low after FTCA and the prophylactic administration of antiemetic drug before anesthesia or after extubation is not necessary.

**Table:** 

Abbreviations, acronyms & symbols	
5-HT3	= 5-hydroxytryptamine type 3
CICU	= Cardiac intensive care unit
FTCA	= Fast-track cardiac anesthesia
PONV	= Postoperative nausea and vomiting

## INTRODUCTION

Postoperative nausea and vomiting (PONV) is defined as any nausea, retching, or vomiting occurring for the first 24-48 hours after surgery in inpatients. The incidence of PONV is about 30% in all post-surgical patients and up to 80% in high-risk patients, it is one of the most common causes of patient dissatisfaction after anesthesia^[1]^. The physiology of PONV is complex and not perfectly understood. The brain structures involved in the pathophysiology of vomiting are distributed throughout the medulla oblongata of the brainstem, not centralized in an anatomically defined center of vomiting^[2]^. Regarding PONV, enterochromaffin cells in the stomach and intestine release serotonin, which binds to 5-hydroxytryptamine type 3 (5-HT_3_) receptors in the gastrointestinal tract? This binding will lead to stimulation of vagal afferents in the gastrointestinal tract that conduct impulses reaching brainstem structures located between the obex levels and the nucleus ambiguus, such as the area postrema. Located on the dorsal surface of the medulla oblongata at the caudal end of the fourth ventricle, the area postrema has a critical role in the central mechanism of vomiting^[2]^. Neurotransmitter receptor systems involved in mediating signals leading to nausea and vomiting include dopaminergic (D_2_), cholinergic (muscarinic), histaminergic (H_1_) serotonergic (5-HT_3_), and neurokinin NK1 systems. The corresponding receptors are potential targets for antiemetic drugs. Even if the numerous neurochemicals involved in the neurocircuitry of emesis may not have been fully identified, the two main inputs to the central pattern generator are from the abdominal vagal afferents via the nucleus tractus solitarius^[3]^ and from the chemoceptive trigger zone located in the area postrema^[4]^.

While studies of PONV have concentrated on non‐cardiac surgical procedures^[5,6]^, limited data suggest that the incidence of PONV in patients undergoing cardiac surgery may be as high as 45-50%^[7-9]^.

Fast-track cardiac anesthesia (FTCA) is a perioperative anesthetic management that aims to facilitate tracheal extubation of patients within 1-6 hours after cardiac surgery. Most centers consider fast-track extubation up to 8-10 hours after surgery^[10]^.

The aim of this study was to evaluate the incidence of PONV after FTCA in the first 24-48 hours in the cardiac intensive care unit (CICU) after open-heart surgery, risk factors for PONV and its influence on CICU length of stay.

## METHODS

In this prospective observational study, we collected data from 300 patients undergoing open-heart surgery from two cardiac centers, Princess Muna Al-Hussein Cardiac Center, King Abdullah University Hospital in the North of Jordan and Queen Alia Heart Institute, Royal Medical Services, Amman, Jordan, over a three years period from the January 1, 2013 to the end of December 2015. These patients underwent coronary artery bypass grafting, valve replacement or combined procedures with FTCA. We did not lose any patients involved in this study, as the mortality was excluded at the beginning of our study. Tracheal extubation performed within 6-10 hours in the CICU when patients were conscious, oriented, with stable hemodynamic status and no bleeding. We did not use routinely nasogastric tube insertion, neither prophylactic antiemetic drug. After completion of surgery, patients were transferred to the CICU, where inotropic, vasodilatory agents were started depending on their hemodynamic status. A light heater was used to ensure the normothermic temperature of the patient. Propofol was immediately used for postoperative sedation (1-4 mg/kg/h) in the first 3 hours, then stopped as spontaneous breathing test and to improve the patient's level of consciousness. After that, analgesia was provided by intermittent boluses according to the patient's condition using low doses of intravenous (IV) morphine (5-15 mg). After 48 hours, we are usually used paracetamol (1 g) IV as analgesia every 6 hours. When patients still complained of severe pain, we used oral tramadol 100 mg tablet(s) twice a day if there was no renal dysfunction, history of peptic ulcer or history of bronchial asthma. The patients were discharged from CICU after 3-4 days, as we do not have a telemetry monitor in the general cardiac ward.

Preoperative (Table 1), postoperative and CICU length of stay data (Table 2) were collected. Test results were considered significant when *P*<0.05. Data were collected after patient and ethical committee approval through The Jordanian University of Science and Technology Institutional Ethics Committee-Jordan-Irbid.

**Table 1:** Preoperative data (n= 300).

Variables	N
Age (years)	Mean age 57.7, ranging from 19-82
Female	112
Male	188
Diabetes mellitus	156
Hypertension	203
Smoking	186

**Table 2:** Postoperative data.

Variables	
Duration of ventilation (hours) - mean (min-max)	5.1 (3.5-12)
Length of stay (days) - mean (min-max)	5 (3-10)
Total morphine consumption (mg) - mean (min-max)	20 (10-30)
Total midazolam consumption (mg) - mean (min-max)	8 (5-10)
Patients with nausea - n (%)	46 (15.3%)
Patients with vomiting - n (%)	31 (10.3%)
Patients who received metoclopramide (Plasil) as first line - n (%)	65 (21.6%)
Patients who received ondansetron (Zofran) as second line - n (%)	12 (4%)

### Anaesthetic Protocol

All patients had a standard anaesthetic technique: fentanyl (10-15 µg/kg) + midazolam 0.05 mg/kg + pancuronium (0.1 mg/kg) for induction, remifentanyl infusion (0.05-2 µg/kg/min) + propofol infusion (50-100 µg/kg/min) + isoflurane (1-2%) or sevoflurane (2-2.5%) for maintenance of anaesthesia. Pharmacological reversal of neuromuscular blockade was not used. Mechanical ventilation was performed through a closed circuit with a tidal volume of 7 mL/kg. Along with the standard ASA monitors (pulse oximetry, electrocardiogram, capnography and nasopharyngeal temperature probe), all patients had an arterial line, central venous line, urinary bladder catheter. Frequent laboratory values for arterial blood gases, blood sugar, hematocrit and electrolytes were obtained. Chest X-ray and echocardiography were done routinely before surgery.

The record of any episodes of nausea, retching or vomiting was started by CICU nurses immediately after extubation and hourly until the end of 48 hours of CICU stay. Because of the fact that retching is similar to vomiting in all aspects except the production of vomitus^[5]^, both were considered as vomiting in the study. Metoclopramide (Plasil) 10 mg IV as antiemetic drug was initially prescribed for all patients by an attending cardiac resident. When there was no response to metoclopramide up to 1 hour and patients still complained of nausea or vomiting, then ondansetron (Zofran) 4 mg IV was prescribed. We did not notice any extrapyramidal symptoms or any other adverse reaction from metoclopramide, and this may be due to irregular use of the drug (single-used, as a single-dose when needed) and not a routine prescription as a regular prophylactic, even with the use of ondansetron. Data were analyzed using the Statistical Package for Social Sciences (SPSS, IBM version 20) and were described using percentages. The differences between percentages were analyzed by the chi-square test. A *P*-value less than 0.05 was considered statistically significant.

## RESULTS

Nausea was reported in 46 (15.3%) patients and vomiting in 31 (10.3%). Among females, 38 (33.9%) patients developed nausea and 20 (17.9%) patients developed vomiting. Among males, 8 (4.3%) patients developed nausea and 11 (5.9%) developed vomiting (Table 3). The rates of nausea and vomiting were significantly higher among females compared to males.

**Table 3:** Incidence of nausea and vomiting after fast-track anaesthesia for heart surgery among males and females.

	Males		Females		Total		*P*-value
	N	%	N	%	N	%	
Nausea	8	4.3%	38	33.9%	46	15.3%	<0.001
Vomiting	11	5.9%	20	17.9%	31	10.3%	<0.001

Sixty-five (21.6%) patients were treated successfully with metoclopramide and in only 12 (4%) metoclopramide failed to manage their symptoms within the first hour of administration, but responded well to ondansetron. We found that female patients and a patient under 60 were more likely to develop postoperative nausea and vomiting, and did not prolong CICU stay.

## DISCUSSION

Nausea and vomiting are unpleasant and common distressing symptoms during recovery from anaesthesia. There is little information on the incidence or management of nausea and vomiting after cardiac surgery although most studies about PONV have concentrated frequently on women undergoing gynaecological procedures^[7]^.

After cardiac surgery, intestinal mucosal hypoperfusion is common and is a known factor that explains the high associated incidence of postoperative nausea and vomiting^[11]^. Recently, it has been demonstrated that the expansion of plasma volume during cardiac surgery is associated with the maintenance of intestinal perfusion and reduction of postoperative morbidity, including persistence of nausea and vomiting^[12]^.

Several hypotheses about the cause of PONV are possible, including prolonged surgery, a large amount of preoperative opioids, and intestinal hypoperfusion in patients without prolonged sedation^[13]^.

Lerman^[14]^ has suggested that the incidence of PONV is approximately 2-3 times greater in women than in men. Women have a 2- to 3-fold greater incidence of PONV than men. This is attributed to increased levels of estrogen, gonadotropin, and plasma progesterone^[15-19]^. Administration of antiemetic drugs to prevent PONV after surgery can be used during, before or after surgery, or to treat well-established PONV. This latter approach is a satisfactory option for patients undergoing surgical procedures with a low frequency of PONV, such as FTCA^[20]^. Although it still provides reasonable control of emesis, the strategy of administering antiemetics as needed may be useful to avoid routine prophylactic administration and avoid the side effects and cost of unneeded antiemetic therapy^[21]^. The role of prophylaxis for PONV in cardiac surgery is still a subject of debate^[13]^. In our practice, we do not use prophylactic antiemetic drugs. The control of emesis by the administration of antiemetic drugs when necessary after extubation in our practice may be the cause of not noticing any extrapyramidal symptoms or any other adverse reaction of metoclopramide or ondansetron. The previous study by Grebenik and Allman^[7]^ and Woodwarg et al.^[8]^ have previously reported a 46-49% incidence of nausea and 37-42% incidence of vomiting in their patients after cardiac surgery. In our study, the incidence of PONV was considerably lower in comparison with their study. This may be related to our anaesthetic protocol, low doses of fentanyl (10-15 µg/kg) and relatively low doses of morphine (5-15 mg) during the postoperative period. Rafiq et al.^[22]^, in their prospective randomized controlled trial evaluating whether a multimodal opioid sparing regimen of dexamethasone, gabapentin, ibuprofen and paracetamol had better analgesic effect, fewer side effects and was safe compared to a traditional regimen of morphine and paracetamol after cardiac surgery. They concluded that, in patients undergoing cardiac surgery, a multimodal regimen offered significantly better analgesia than a traditional opiate regimen. Complaints of nausea and vomiting were significantly reduced. No safety issues were observed with the multimodal regimen. In our study, we observed a relatively low incidence of PONV with single regimen, our anaesthetic protocol was relatively well followed, using low dose of morphine (5-15 mg) during the postoperative period, safely and without complications. The argument against the use of prophylaxis may be due to the fact that droperidol and ondansetron may be associated with cardiac arrhythmias^[13]^. The use of prophylaxis, as we mentioned before for PONV in cardiac surgery, is up for debate, following a report from a study showing a considerably lower prevalence of nausea (19.7%) and vomiting (4.3%) after cardiac surgery with fast-track anesthesia when effective rescue treatment was implemented, leading the authors to discourage the use of prophylaxis^[20]^.

The Cochrane database systematic reviews, by Wong et al.^[23]^, sought to determine the safety and effectiveness of fast-track cardiac care compared with conventional (not fast-track) care in adult patients undergoing cardiac surgery. Fast-track cardiac care intervention includes the administration of low-dose opioid-based general anaesthesia or the use of a time-directed extubation protocol, or both. This is an update of a Cochrane review first published in 2003^[24]^, updated in 2012^[25]^ and updated again in 2016^[23]^.

They searched the Cochrane Central Register of Controlled Trials (CENTRAL; 2015, Issue 5), MEDLINE (January 2012 to May 2015), Embase (January 2012 to May 2015), the Cumulative Index to Nursing and Allied Health Literature (CINAHL; January 2012 to May 2015) and the Institute for Scientific Information (ISI) Web of Science (January 2012 to May 2015), along with reference lists of articles to identify additional trials. They did not apply language restrictions. Their analysis of the Cochrane database systematic reviews included all randomized controlled trials of adult cardiac surgical patients (coronary artery bypass grafts, aortic valve replacement, mitral valve replacement) that compared fast-track cardiac care and conventional (not fast-track) care groups. They focused on the following fast-track interventions, which were designed for early extubation after surgery: administration of low-dose opioid-based general anaesthesia during cardiac surgery and use of a time-directed extubation protocol after surgery. The primary outcome was a mortality risk. Secondary outcomes included postoperative complications, reintubation within 24 hours of surgery, time to extubation, length of stay in the intensive care unit and in the hospital, quality of life after surgery and hospital costs. They concluded that low-dose opioid-based general anaesthesia and time-directed extubation protocols for fast-track interventions have mortality risks and major postoperative complications similar to those of conventional (not fast-track) care, and therefore appear to be safe for use in low- to moderate-risk patients. These fast-track interventions reduced time to extubation and shortened the length of stay in the intensive care unit, but did not reduce the length of stay in the hospital.

A specific PONV risk assessment tool may be useful for predicting those at higher risk after cardiac surgery. Further research is required to identify strategies to reduce PONV^[26]^.

### Limitation of the Study

Although this is a prospective observational study of two cardiac centers with the same anesthetic protocol, a prospective, randomized, double-blind study with single-center experience, involving a greater number of patients with a standardized anesthetic protocol, even with different surgeons, will be more valuable and we think will be of value to consider a prophylactic approach beside side effects and costs, taking into account the patient satisfaction. It will be of value to test groups of patients of different age and sex, in a randomized double-blind study involving both groups of patients younger than 60 years of age, male and female, with groups of patients over 60 years old.

## CONCLUSION

In our study, the incidence of PONV is relatively low after cardiac anesthesia. We found that female patients, younger than 60 years, were more likely to develop postoperative nausea and vomiting, and did not prolong CICU stay. Control of emesis by the administration of antiemetic drugs when necessary after extubation will eliminate the side effects and the cost of unneeded routine prophylactic antiemetic drugs.

**Table:** 

Authors' roles & responsibilities	
EMH	Substantial contributions to the conception or design of the work; or the acquisition, analysis, or interpretation of data for the work; drafting the work or revising it critically for important intellectual content; agreement to be accountable for all aspects of the work in ensuring that questions related to the accuracy or integrity of any part of the work are appropriately investigated and resolved; final approval of the version to be published
HE	Substantial contributions to the conception or design of the work; or the acquisition, analysis, or interpretation of data for the work; drafting the work or revising it critically for important intellectual content; agreement to be accountable for all aspects of the work in ensuring that questions related to the accuracy or integrity of any part of the work are appropriately investigated and resolved; final approval of the version to be published
NAZ	Substantial contributions to the conception or design of the work; or the acquisition, analysis, or interpretation of data for the work; drafting the work or revising it critically for important intellectual content; agreement to be accountable for all aspects of the work in ensuring that questions related to the accuracy or integrity of any part of the work are appropriately investigated and resolved; final approval of the version to be published
HR	Substantial contributions to the conception or design of the work; or the acquisition, analysis, or interpretation of data for the work; drafting the work or revising it critically for important intellectual content; agreement to be accountable for all aspects of the work in ensuring that questions related to the accuracy or integrity of any part of the work are appropriately investigated and resolved; final approval of the version to be published
